# IoT Micro-Blockchain Fundamentals

**DOI:** 10.3390/s21082784

**Published:** 2021-04-15

**Authors:** Aristidis G. Anagnostakis, Nikolaos Giannakeas, Markos G. Tsipouras, Euripidis Glavas, Alexandros T. Tzallas

**Affiliations:** 1 Department of Accounting and Finance, University of Ioannina, GR48100 Preveza, Greece; 2 Department of Informatics and Telecommunications, University of Ioannina, GR47100 Arta, Greece; giannakeas@uoi.gr (N.G.); eglavas@uoi.gr (E.G.); tzallas@uoi.gr (A.T.T.); 3 Department of Electrical and Computer Engineering, University of Western Macedonia, GR50100 Kozani, Greece; mtsipouras@uowm.gr

**Keywords:** autonomous blockchain, micro-blockchain, microcontroller blockchain, IoT blockchain, peer IoT networks, smart dust blockchain

## Abstract

In this paper we investigate the essential minimum functionality of the autonomous blockchain, and the minimum hardware and software required to support it in the micro-scale in the IoT world. The application of deep-blockchain operation in the lower-level activity of the IoT ecosystem, is expected to bring profound clarity and constitutes a unique challenge. Setting up and operating bit-level blockchain mechanisms on minimal IoT elements like smart switches and active sensors, mandates pushing blockchain engineering to the limits. “How deep can blockchain actually go?” “Which is the minimum Thing of the IoT world that can actually deliver autonomous blockchain functionality?” To answer, an experiment based on IoT micro-controllers was set. The “*Witness Protocol*” was defined to set the minimum essential micro-blockchain functionality. The protocol was developed and installed on a peer, ad-hoc, autonomous network of casual, real-life IoT micro-devices. The setup was tested, benchmarked, and evaluated in terms of computational needs, efficiency, and collective resistance against malicious attacks. The leading considerations are highlighted, and the results of the experiment are presented. Findings are intriguing and prove that fully autonomous, private micro-blockchain networks are absolutely feasible in the smart dust world, utilizing the capacities of the existing low-end IoT devices.

## 1. Introduction

The IoT is expanding rapidly, based on billions of low-cost, low-end micro-devices [1]. Establishing blockchain operations on every level in the IoT ecosystem is speedily becoming a field of intensive research activity, due to the unique operational clarity and collective immunity it delivers [2].

Remarkable works in the field are coming to the light of publicity. In [3], a nonrepudiation blockchain architecture for industrial IoT applications is presented by Yang et al., while in [4] an arbitrable data auditing blockchain-based schema for running a secure storage-as-a-service network over the cloud is presented. The ΙΟΤΑ cryptocurrency framework for sharing value in the IoT world through the Tangle IoT network is introduced in [5], while in [6] a private, domain specific PoS architecture for immune, structured data sharing over the cloud is analyzed. Most of these works are presenting comparatively complex blockchain architectures of the “cryptocurrency type” that are built to operate on the “upper-levels” of IoT ecosystem. With a few exceptions like in [7], they are considering the presence of plentiful, in the sense of “always adequate” processing capacity, memory, power and network resources on the nodes. Still, the low-cost low-level IoT components of the smart dust world such as active sensors and smart switches [8], are characterized by extremely minimal capacities and operability.

The primary objective of the current work is to highlight “minimum”. This paper, attempts to answer to the questions: “How *deep* can a blockchain go?”, “How much of a *thing* must an IoT component be to be able to engage in peer, autonomous, blockchain operation?”

To establish autonomous, end-to-end blockchain operations in the IoT world, we need to “plant” it “as deep as possible” inside the tiny IoT devices. We need to get down to single-processor, single-sensor, even single-accumulator level. In the quest to reach the limits, blockchain components must be analyzed in terms of “glass-box”, and to be constrained down to the absolutely necessary. This will enable every minimal IoT device, even the “hard-wired” ones like smart switches and active sensors, to spontaneously form and operate autonomous micro-blockchains.

The proposed *Witness Protocol* is defined as a “minimal functionality containment”, to operate the minimum essential autonomous blockchain on the least-possible hardware configuration. In our experiment the *Witness Protocol*, is utilized to establish minimum blockchain functionality over a network of IoT microcontrollers. The protocol resembles to the “ear-witness” real world scenario. It creates and runs a *minimal*, *distributed*, blockchain structure, over an *ad-hoc local network neighborhood* of *peer*, *autonomous* microcontrollers, without the prerequisite of additional equipment or centralized network access. The protocol is designed to meet to the features and the limitations of the conventional IoT devices. It utilizes their limited memory and computational capacity to build an overall *collective resistance* to malicious attacks, which is “significantly large” in comparison to that of the isolated IoT device.

Introducing blockchain to the lowest operational level of the IoT world, will bring unprecedented transactional clarity and immunity to malicious attacks. It is a rational step towards a “world of absolute trust”, a world where questioning “done” becomes meaningless. Even the tiniest actions, like turning on the light, opening a door, tapping on a screen, or accessing a data record, can become provable and verifiable in an undoubtable manner on a collective basis. Being for example able to verify without doubt *when car was last serviced*, *a door was unlocked* or *what was the temperature reading inside a refrigerator*, can become of vital significance, even in casual, “non-military” environments (especially under the scope of the recent pandemic).

In contradiction to the “cryptocurrency” type of protocols [9], in which traversing the entire blockchain back to the constitutional *Block 0* is absolutely necessary, the *Witness Protocol* defines essential nonrepudiation functionality on “*limited-horizon events’ history*”, over *partial symphony* and *eventual consistency* environments [10,11].

Our experiment proves in practice that micro-blockchains of basic functionality can be well-run in the micro-controller level on the current IoT devices. Getting even deeper, down to the single-switch or single-sensor level is considered *feasible* under the assumption that the components *are active*, they can perform a *minimum amount of actions* even in “hardwired” implementation and they have sufficient memory to store at least *two consecutive transactions’ data*. The *Witness Protocol* is developed and tested on Arduino Nano 33 IoT microcontrollers, which proved capable of processing everyday transactions in “human timescale” event frequencies (i.e., $10^{-5}\sim 10^{2}\;\mathrm{Hz}$), over neighborhoods of 10s of IoT devices. The system is benchmarked and evaluated in terms of *neighborhood population*, *computational efficiency*, and *resilience to malicious attacks*. As part of the ongoing research, the setup will be utilized for immunizing data readings form sensors in the field of healthcare.

## 2. Background

Establishing micro-blockchain operation in autonomous networks of peer IoT micro-devices requires thorough consideration of the technological features of the IoT devices, as well as of the conceptual frontiers of the blockchain models. How exactly, and why should blockchain apply in the low-level IoT? What can it improve and on what cost? Which are the minimum technical requirements for this?

Blockchain was primarily developed to bring trust in cryptographic currencies’ transactions and is often “miss-classified” under this prism. Still, its functionality can extend way beyond. The principles of blockchain can be applied on any system of peer, transacting entities, to deliver collective immunity and trust.

On the other hand, the majority of the IoT micro-devices are designed and utilized to carry out mostly trivial, automated tasks, such as gathering and sharing data from their environment. In many applications they constitute the physical “access points” of the cloud to the real world. They bear “*by definition*”, significant limitations in *memory capacity, processing power*, *consumption*, *networking*, and *data transfer capabilities* in comparison to the high-performance nodes. Many of them, like for instance *PLC’s* and *microcontrollers*, allow only for minimal, single-threaded code execution, while others, like *i-beacons* can merely be classified as programable machines. Identifying the minimum requirements for blockchain operation on smart dust micro-networks is essential.

Micro-networking has been the trend for several years; autonomous IoT devices, capable both in forming peer micro networks, as well as in connecting broadly over the internet are becoming mainstream. Evolving standards, such as the *Bluetooth-mesh*, which enable powerful concurrent multicast many-to-many communication in networks with thousands of devices, the *Bluetooth Low Energy*, *NFC*, and *LoRa WAN* provide IoT devices with significant communication capabilities [12]. Extremely low-consumption devices like *i-beacons* and *RF-ids*, demonstrate in practice that the micro-networking era has arrived and is here to stay [13].

Technologies in the world of micro-devices are becoming mature enough to support sophisticated internetworking, providing the ideal testbed for new ideas.

### 2.1. What Is a Blockchain Made of?

Taking blockchain back to its “primevals”: The primitive notion upon which the total construct of “universal trust” relies, is the consistent recursive application of a single-direction, monotonical transformation, over a time-evolving sequence of data [14,15]. Blockchain is in essence the combination of two major logical mechanisms:

- The hashing mechanism, which binds the chain links together, and
- The consensus mechanism, which safeguards the integrity of the chain throughout the network [16].

Peer-ness also constitutes a mandate: with the penalty of exclusion, every node in the Blockchain network is operating using the same hashing and consensus algorithm; no one can violate the rules and keep being part of the community.

#### 2.1.1. The Hashing Mechanism

Secure hashing algorithms are information reduction mechanisms, producing digital “fingerprints” of the input data. Hashing fingerprints are a “few bits” words. The process is lossy in terms of information and, there is no effective way of acquiring the initial data from a hash sequence; tiny variations on the initial message (e.g., 1-bit) results to a chaotically different hash (counter-example: the homomorphic hashing, that clusters hashes in response to clustered inputs). The process is susceptible only to brute force attacks. It is estimated that a brute force attack over a 64 bytes long initial message which is hashed with the SHA-256 algorithm would require 36~64 years at a 1 M/s attempts rate utilizing every available CPU in the world [17]. Bringing blockchains to the lowest operational level, requires efficient cryptographic hash function implementations, both in terms of energy consumption, as well as in terms of computational and communications’ load.

The hash chain is the primitive mechanism for creating blockchain structures. In this, each node is formed by the successive application of a cryptographic hash function h to an initial string x. h(h(h(h(x)))) gives a hash chain of length 4—often denoted h^4(x). Bitcoin blockchain is reliably operating on the SHA-256 algorithm, providing a solid proof of the concept: any device capable of implementing the robust SHA-256 algorithm, can produce profoundly hard to compromise hash chains.

Another structure commonly utilized in blockchains is the Merkle tree [18]. Merkle trees are hash trees in which the leaves contain the actual data, and the non-leaf nodes are hashes of their children.

Multiple input strings describing concurrent transactions are combined in Merkle trees through recursive hashing. It is straightforward and computationally inexpensive for the recipient of the data to verify their correctness, if he only knows the “essential node values” of the Merkle tree (in the generic case of a complete binary Merkle tree, he has to know the root and a minimum of $log_{2}\left(m\right)-1$ nodes, where m is the total number of the leaves). Merkle trees are utilized in blockchain to cope with high transaction rates. For our purposes, they can only be considered for application in “higher-end” IoT micro-nodes, with increased memory and processing capacity (e.g., *Raspberry Pi™*).

#### 2.1.2. The Consensus Mechanism

The consensus amongst the blockchain nodes is fundamental. In public PoW chains [19,20,21] such as the bitcoin [22], the consensus-building process is effectively “hardwired”. In the smart contracts’ world [23,24], building consensus becomes extremely flexible and allows for alternative consensus proofs like the *Proof of Stake* or the *Proof of Authority*. Still, not every blockchain implementation shares the same needs: the mechanisms for building consensus, range from “hardwired” (as in bitcoin) to “totally programable” (as in smart contracts).

### 2.2. Consistency over Distributed Networks

*“In an ideal world there would be only one consistency model: when an update is made all observers would see that update.”* [25].

The CAP theorem, defined in [26], suggests this is not feasible in the generic case. According to it, out of the three major properties of shared-data systems (i.e., *data consistency*, *system availability*, and *tolerance to network partition*) only two can be satisfied at the same time.

Perhaps the most treasured property in the IoT world is *system availability*: “*I should always be able to unlock my car, in spite of the network existence or status*”. Under this directive every *Local* Event must be treated by the local node in absolute priority. In addition, keeping local storage consistent is also mandatory in most of the practical IoT applications. “*The last unlock of my car has always have to be recorded locally and be known as last*”. To satisfy this, the system must facilitate *Monotonic Writes*.

#### Strong, Weak and Eventual Consistency

*How much “consistency” can be supported in the IoT micro-scale?*

Throughout the literature, three types of data consistency are identified:

- Strong consistency: In the light of an update anywhere in the network, any subsequent access will return the same result (i.e., the most recent).
- Weak consistency: There is no guarantee that all processes following an update will return the same value all the time; in fact, a number of conditions has to take place for this to happen.
- Eventual consistency: A special case of weak consistency, in which the notion of a finite duration “inconsistency window” is introduced. A number of eventual consistency attributes are identified, namely *Casual consistency, Read your last write, Monotonic Reads and Writes.* More than one of these properties may be combined in a distributed system. Eventual consistency is an elegant trade-off, which, tailored accordingly, can deliver the conceptual framework for requirements to became satisfied by a system.

Eventual consistency models can be fine-tuned to meet the system requirements on each application.

### 2.3. IoT Scale Boundaries

As already mentioned, applying blockchain in the micro-scale, requires micro-devices to setup and operate blockchain mechanisms at the minimum possible resources. Due to the inevitable memory constraints, they must be able to operate over a finite, minimal “historical events horizon”.

#### Effective Events Horizon

Even though in the generic blockchains of “*cryptocurrency type*” the entire records back to “*Block 0*” has to be constantly traversed, not every application shares the same *prerequisite of infinity*. In many cases, going “all the way back” is not an absolute necessity. Even a Limited historical horizon of recorded events can prove enough to increase the overall robustness. In fact, any amount of local memory capable of storing *two or more consecutive transactions* induces positive impact to the collective immunity. The notion of *partial synchrony* becomes essential.

Additionally, in the generic case it is not even feasible to store the entire blockchain in every micro-device at all time. To not compromise the requirement for ad-hoc, peer and autonomous operation, efficient policies must be adopted.

**Figure 1:**
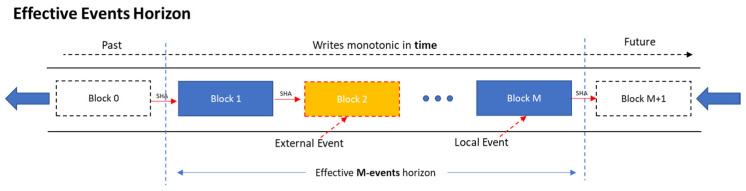
IoT effective events horizon modeled as a FIFO Hash Chain.

**Figure 2:**
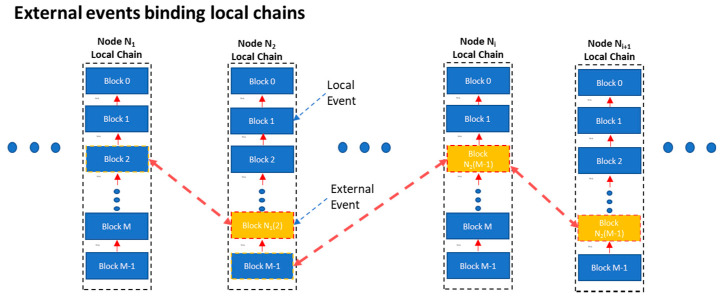
Micro-blockchain as a sparse aggregate of local chains.

Every IoT device can store in its’ memory a maximum of M data blocks, representing the M most recent transaction events. These constitute the “Effective events’ horizon” of the device. The process can be modeled as a fixed-size FIFO Hash-chain, depicted in Figure 1:

In addition, most IoT devices lack a real-time clock. In the proposed *Witness Protocol*, time is defined relatively. Events from neighboring devices (*External Events*) are stored in the hash chain along with events that take place locally (*Local Events*). Each event taking place in a device (node) is monotonically recorded and acts as a relative timestamp to the local chains of the receiving nodes. The entire micro-blockchain can then be seen as the construct (aggregate) of the collective memory of all nodes, depicted in Figure 2:

Keeping a mirror-copy of the entire activity on every IoT device in every other is not feasible in the generic case, and poses prohibiting memory and communication overheads. Under the scope of the CAP theorem (discussed earlier), raising a hard requirement for mirror redundancy would certainly jeopardize node availability. More flexible approaches are feasible, (as for example the Probabilistic Finality paradigms discussed in [27]). In our implementation, every IoT node stores only part of the entire chain, (aka its’ own “view” of the entire chain).

The operation of the IoT micro-devices is characterized by Increased locality of references in *time* and *space*. This allows for a highly efficient utilization of the resources. In the case of “*extremely limited memory capacity*”, which may practically scale down to few-bit accumulators, the effective *horizon of events* of a device can become accordingly short; still, even the tiniest capacity, can become substantial part of the ecosystem, providing valuable validation data to siblings and increasing the overall robustness.

## 3. IoT Micro-Blockchain Essentials

The *Witness Protocol* introduced in this work, defines the functionality of a model-blockchain, minimal enough to operate in the minimum imaginable scale within the IoT micro-devices. We address this model under the graceful term “micro-blockchain”.

### 3.1. Unilateral Transactions

The usefulness of micro-blockchain extends way beyond modeling monetary transactions; the nature and the essence of *transaction* can be extended beyond the classic bilateral act of “give and take”. In fact, *transaction* can be semantically reduced to the bit-level, to capture a single toggle of an electric switch. For instance, unlocking a secured door, turning on a light, or even accessing a specific bit of data, are *unilateral transactions*, that do not necessarily invoke more than one actors; still they constitute *local events* and raise the need for being recorded in blockchain.

Extending the notion of *Transaction* to the space of *unilateral actions* is essential; it constitutes the philosophical cornerstone for the application of the fundamental blockchain initiatives in every single action in the IoT world. Unilateral actions are themselves parts of the whole, “capable” of altering the current state of the collective system memory.

### 3.2. Locality of Events

In the IoT world, extensive *locality* is observed both in time and space; under the scope of this paper, we use blockchain as a robust construct for the provision of a *distributed*, *immune* mechanism, upon which *local transactions* can be inter-*Locked* and intra-*Verified* over local autonomous networks. As described in the previous sections, for a linked list to become blockchain, at least two semantic mechanisms must exist simultaneously, the *hashing mechanism for linking the records* and the *consensus mechanism for allowing verification*. Following, we shall try to set a minimum requirements threshold for these.

- **Assumption A:** If a device can perform hashing, then it is capable of building a local hash chain.

Merkle trees may be also developed on top of the local hash-chains, to cope with increased events rate occurrence on a device and to further augment “internal” immunity. Due to the fact that they introduce additional write cost, computational complexity and storage needs, they are not investigated in this work.

- **Assumption B:** Building consensus requires knowledge of the domain and the frame in which the Blockchain is going to operate.

The *Witness Protocol* is mainly defined to raise collective non-repudiation and follows a “write-intensive” consideration. *Writes* are expected to occur at relatively high rates, while *verifications* will only occur in the light of external investigation (dispute resolution) or apparent inconsistency.

Consensus is then achieved under two “low-cost” presumptions:

- Every *Node* running the same code (implementing the *Witness Protocol*) is trusted trustee (unless proven false).
- A *Verifier* can access concurrently the memory of every node in the network. In practice, every node with adequate storage capacity may act as verifier; still for the purposes of our experiment, we consider the verifier to be an abstract entity with concurrent access in the “collective memory” of all nodes.

### 3.3. Collective Memory

Treasuring the peer-ness and autonomy of the IoT world, the “absolute syncing” demand must be relaxed: not all nodes have to be aware of the entire chain. In the *Witness Protocol*, every node is mostly occupied handling its’ “*own events*” and spends a significant amount of its “*free time*” to listen to its’ *Neighbors*. As soon as an incoming external event report is captured, the local node verifies it and adds it into his chain—thus building its’ own version of the *universal truth*. It is the aggregate contents of the memories that compose the entire Blockchain. The construct is susceptible only of 51% attacks.

In the generic case, the entire data sequence may not be “recoverable” at all time. In the light of *absolute coherency* requirement, the collective memory may be viewed as a collective Redundant Array of Inexpensive Disks (RAID).

### 3.4. Minimum Hardware Requirements

We will now try to answer to the question: How much of a “thing” must an IoT device be to be able to engage spontaneous micro-blockchain activity?

To autonomously operate in a peer, elementary chain, a general purpose IoT device must be “at-least” capable of:

(a) Defining primitive data structures (to represent transactions’ data)
(b) Storing at minimum two consecutive transaction records
(c) Binding the records within hash chains (done through secure hashing)
(d) Signing the records (done through public key cryptography)
(e) Transmitting & receiving data to and from other alike devices within its’ scope/reach

**Figure 3:**
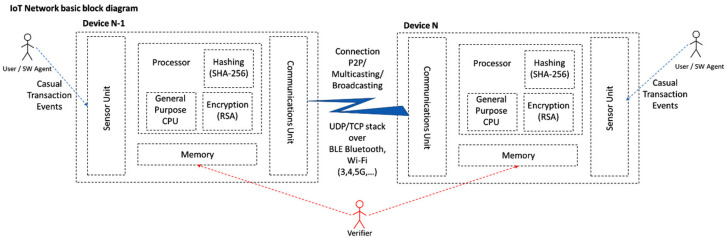
Elementary IoT device blockchain-capable configuration block diagram.

The minimum ingredients to build an elementary chain are presented in Figure 3:

Prerequisites (a), (b), (c) and (d) proclaim the convenience of the IoT device being a Turing machine, capable of executing general purpose programs. In addition, the existence of a “Turing complete” code execution machine, theoretically allows for smart contract execution-capable blockchains, operating in pure-peer-mode in the IoT world. The existence of a minimal programmable framework is also a very convenient approach while developing and testing protocols. Still, general purpose “Turing” programmability is not compulsory to establish micro-blockchain; the required functionality can be hardwired, leading to even lower capacity, special-purpose smart dust hardware.

IoT devices are designed to cope with real-world conditions and are often used for real-world interfacing, like temperature measurement, doors’ unlocking, etc. This poses a rational minimum limit in IoT micro-blockchains’ performance: they must be at-least capable of operating within the human-perception timescale. In the vast majority of the cases, the rate of the recordings spans in the range of 10^−5^~10^2^ Hz (from a few hundred events per second to some events per hour).

In the generic case, enough CPU power to conduct the primitive hashing and signing functions (i.e., SHA-256 & RSA in our experiment) is necessary [28,29]. In addition, enough memory to store the program and at least two consequent event records in the local chain is needed as well. Even though the system becomes *multivariate* (as discussed in the Results section), indicative performance outlines for the *Witness Protocol* are presented in Table 1:

### 3.5. The Experiment: Smartdust Blockchain

In our ground-base scenario, *N* autonomous, peer, IoT microcontrollers operating in a *Neighborhood* of peer devices are forming the micro-blockchain, providing the conceptual framework to our study. The objective of the chain is to provide with a robust “Witness/Guarantor” mechanism: Upon the advent of a local event (e.g., a door unlocking), *W* randomly picked neighbors are becoming potential “external” witnesses.

We define *Neighborhood* as a cluster of IoT devices, which fall into the communication range of each other. Even though in our experiment the devices were located in Wi-Fi range from each other, the intra-node communication takes place over TCP/IP, suggesting that any internet capable device can be part of the neighborhood irrespective of its geo-location.

**Figure 4:**
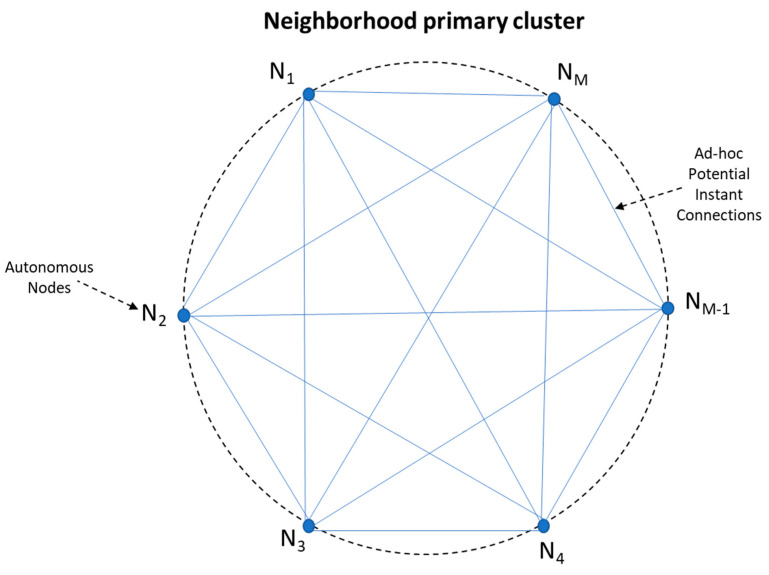
Neighborhood of nodes as a graph.

Neighborhood may be modeled as an evolving complete graph, devices (Nodes) being the vertices and instant point to point communication paths the edges. In Figure 4 we depict a neighborhood of nodes, as a graph. *Neighborhood* is dynamic and new Nodes can join in “ad-hoc”: every device sharing the same source code (i.e., implementing the *Witness Protocol*) can freely connect to and disconnect from any other node.

All nodes are minimal, autonomous, peer components, capable of connecting directly to each other, running copy of the same source code.

### 3.6. Definitions

*Node*: the primary autonomous IoT micro-device. Each device primarily exists to carry out some transaction such as a door lock/unlock, and secondarily to keep a “log record” of its’ activity. All nodes in the neighborhood are trusted trustees, communicating on P2P mode.

*Directive 0*: Nodes primarily exist to serve events happening locally (aka “*Local Events*”), like the the push of a button, the reading of a sensor etc. In this paper we address this requirement as “*Directive 0*”.

*Directive 1*: The nodes secondarily exist to store in their memory a “monotonic log” of the *Local Events*, and to notify their neighbours of them. We call this attribute “*Directive 1*”.

*Community*: The superset of all devices implementing the *Witness Protocol*

*Neighborhood*: Dynamic construct, representing the casual set of devices in the communication space each time.

*N*: the set of nodes in the neighborhood at a given time

*M*: the (maximum) length of the local chain on each node counted in number of blocks -subject to micro-devices’ memory capacity. In our experiment we consider that all nodes in the neighborhood have the same storage capacity (i.e., *M* events).

*W*: the set of nodes witnessing an event (*Witnesses*)

*T_effective_*: the average new-event occurrence period in a node

*Local Event*: Any “worth storing” event (e.g., the push of a button)

**Figure 5:**
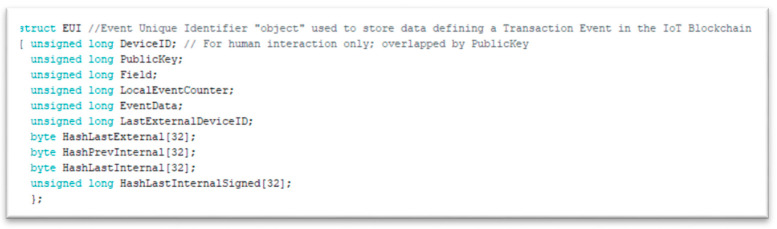
The event unique identifier (EUI) structure.

*Event Unique Identifier (EUI)*: The main block structure, a collection of data identifying an *Event* in a universally unique way throughout the *Community*. The structure of the actual event unique identifier of our experiment is illustrated in Figure 5:

*External event*: “Other nodes’” event witnessed by Node N in its’ Local Chain.

Events are created in the same average rate on every node in a neighborhood; no “*greedy*”, nor “*lazy*” nodes are considered in this study.

### 3.7. The IoT Witness Protocol

The *Witness Protocol* introduced herein is implementing an *Open* blockchain operating in *Private* mode. It is designed to safeguard the consistency in the recordkeeping process:

*“IF an event takes place in node N_i_ which is member of the witness Community,*

*THEN the Community can verify that node N_i_ reported it did,*

*in an -exceptionally hard- to question manner”.*

The idea is simple: every (last) local transaction which takes place on a device is being stored locally and broadcasted to a set of W randomly selected neighbors. Every peer receiver (witness) uses this as an external “lock”, on its’ local chain. Each device can store the last M transactions data (M > 2). Upon this FIFO queue of events, a Merkle tree can be built to cope with high-rate bursts of local events. The overall schema falls to the super-category of *Rendezvous distributed hashing* [30].

#### 3.7.1. Considerations

1. Open, Private mode of operation: any IoT device can become casual node, by simply implementing the *Witness Protocol*.

The *Witness Protocol* facilitates “open and private” blockchain operation. Nodes are self-contained and self-acting, choosing to transmit and to accept data “at will”. They autonomously choose if they want to transmit data, and who their witnesses would be. The transmission is instantiated by the “data owning” device, to a selected set of w peers, building collective non-repudiation immunity. All potentially sensitive data transmitted are RSA-encrypted. Blockchain comes on top of the casual communication process to facilitate an extra level of resistance to malicious attacks.

2. Implied consensus: at the system launch, all nodes implementing the *Witness Protocol* are considered “*trusted-trustees*”. Only a *Reader/Verifier* with access in all nodes can tell malicious nodes through majority.
3. The *Reader/Verifier* can access the memory data of all nodes in range.

The *Witness Protocol* implements an eventual consistency mechanism and quests its conceptual limits: the overall “reality” of the system being the aggregate of the local memories, each IoT device holds a highly “personalized” view. The micro-blockchain can be modeled as a multi-graph of interlinked chain-lists. Each list is evolving monotonically in time, and external events stored in it act as relative timestamps to the overall construct, since, in the generic case, IoT devices do not bear world time clock functionality.

#### 3.7.2. Basic Flow

Each node is *always* in one of two states:

- “*Listener*”: This is the default mode. If there are no new *Local Events* to process, the node is waiting its’ neighbors to connect and pass their latest event data.
- “*Teller*”: Upon the rise of a new *Local Event*, the node enters the “*Teller*” mode. It processes the event data, stores it locally and broadcasts it to its’ neighbors. After this, it returns to the “*Listener*” mode, until a new local event takes place.

In more detail, upon the advent of a new *Local* Event, in *Node_i_*, it:

1. Stores it to its local memory.
2. Contacts *W* (randomly picked) neighbors to inform them of the event. Irrespective of their availability, N returns in “*Listener*” mode after a number of attempts. This way *Node_i_* continues to satisfy *Directives 0* and *1*, even in the case of unavailability of some or all the *W*. This way the system stabilizes. In the opposite case, every Node could eventually become “trapped” trying to contact witnesses and the system would be destabilized in an “all transmitting” condition.
3. Falls back into “*Listener*” state when *write* is completed.

#### 3.7.3. The Write Process

Each node $N_{i}$ stores events on its’ local chain monotonically in time. Apart from data related to the local activity taking place in $N_{i}$, (e.g., “*door unlocked*”) each block also contains the hash of the latest *External Event* it has ever witnessed. External events are added upon the request of sibling neighbors: $N_{i}$ verifies the validity of the received event data (EUI) and stores it in its’ local chain. External events recorded on the local chain, act as “community collective keys” binding all local chains together.

**Figure 6:**
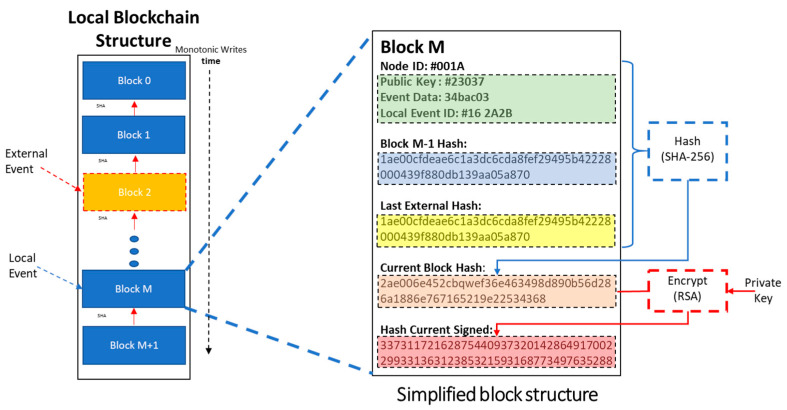
Local chain structure.

The model of the EUI is depicted in Figure 6.

Ideally, we would like W=N−1, i.e., all neighboring nodes witness a local event taking place on $N_{i}$. Demanding every node to witness every local event taking place in every other node in the neighborhood, resembles to “*full-database replication*” scenario. As we have seen earlier, according to the CAP, this cannot be done without sacrificing *system availability*, or *tolerance to network partition,* and thus in the generic case the system is expected to perform with W<N−1. It is reminded that the *Witness Protocol* is designed to optimize *Writes*. Any number of witnesses W>0 has an overall positive impact, increasing the collective non-repudiation resistance in comparison to the isolated node “local only” hash-list storage.

**Figure 7:**
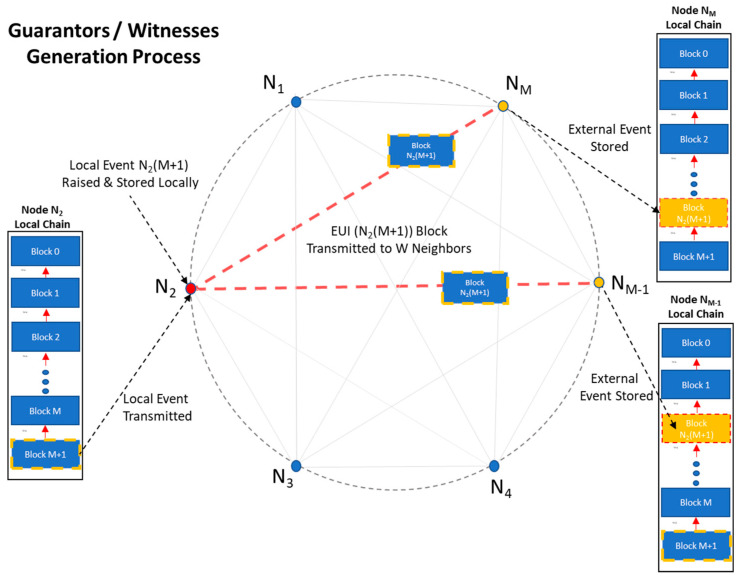
Transmitting local events.

The process of event transmission is depicted in Figure 7.

Writes are conducted in linear *O(1 + W)* cost. To optimize collective immunity against *false witness* attacks, *W* has to be the largest possible; the optimal achievable value of |W| is subject to the system characteristics (i.e., *Events’ rate and distribution*, *Communication latency*, *Energy consumption, Number of nodes in Neighborhood*, *Preemption strategy*) and is concluded accordingly. In Section 4 Results, the way *W* affects (and is affected) by the other characteristics of the system is highlighted.

IoT devices’ limited storage capacity suggests that event records are accessible/verifiable in a finite event horizon. It is reminded that, in the generic case, IoT devices do not bear internal world-time clock and relative synchronization is achieved via the monotonic write property of the devices. Absolute world-time stamping therefore is feasible only if a node (or a set of nodes) capable of world-time timekeeping comes to the neighborhood. Every new event creates a repeated one-hope “ripple” without “gossiping” or repeating past events.

#### 3.7.4. Definitive Decisions—Eventual Consistency

As discussed earlier, every node stores only a part of the entire chain. Still, we shall demonstrate that the *Witness Protocol* delivers substantial immunity. In terms of collective storage, in a stationary neighborhood the *Witness Protocol* resembles to a collective RAID. The system can tolerate an overall total failure of $\frac{W}{2}-1$ nodes without data loss and without compromising its’ event verification ability. In the generic “ad-hoc neighborhood” though, full recovery and verification of every transaction in the horizon of events cannot be guaranteed for small values of *W*. A modest prerequisite is that a kernel of more than |W| nodes remain “stationary” within the average events horizon of the system.

#### 3.7.5. The Read/Verification Process

The *Witness Protocol* aims in increasing the collective non-repudiation immunity. An event is considered verified, if at least $1+\frac{W}{2}$ nodes verify it. In a way analogous to real life “ear-witness” or “loan-guarantor”, even a single casual witness/guarantor drastically improves the system robustness. In practice, *W* may change overtime to cope with the varying *N* and events rate. In our post processing model, we study the behavior of the *Witness Protocol* for *W* varying from 1 to *N*.

To satisfy *Directive 0*, the proposed architecture optimizes *writes*. The tradeoff raises inevitably: the verification process requires full access on every local chain, raising an average event verification cost of $O\left(N*\frac{M}{2}\right)$.

### 3.8. Methodology

**Figure 8:**
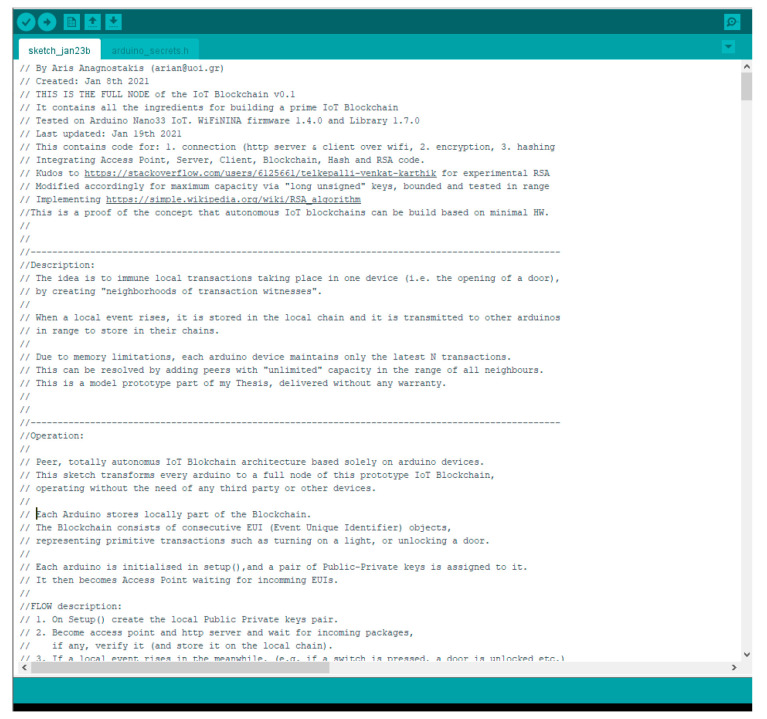
Sketch v.1.8.13 Arduino IDE (Source files @: https://github.com/arianagnostakis/IoT_Blockchain, accessed on 2 February 2021).

The experiment was divided in two phases:

- Phase A: The code implementing the *Witness Protocol* was developed and debugged on the Sketch v.1.8.13 Arduino IDE (Figure 8). The selected target machine was Arduino Nano33 IoT. The details of the software modules and the testbed are discussed below.
- Phase B: The code was physically installed, ran, and benchmarked on a setup of two microcontrollers. Several iterations have been carried out over a 40-day period. The observations led the construction of a numerical “post-processing” model in Octave 6.1.0. The benchmarks were then fed to the module, to study the behavior and the scale-up of the solution in different scenarios, for varying number of nodes, witnesses and event rates.

The results are presented and discussed on Section 4 and Section 5, respectively.

#### 3.8.1. The Ingredients

In Section 3.4 the basic components needed to build a *Node* are identified (i.e., a hashing mechanism, a public key encryption mechanism, a way to communicate to each-other).

Each node operates per case as: *Access Point*, *Server*, *Client*, *Hash & RSA Encoder and Verifier*, and *Storage*. In our experiment we developed, integrated, and tested code for building an access point, a server, a client, secure hash and encryption functionality.

- *Public Private Key Infrastructure*: 32-bit RSA is chosen as the PPK infrastructure. The library provided in [31] was debugged, augmented to 32-bit <long unsigned> and tested. On Setup() a pair of public/private keys is created. The public key of the node acts as Public ID for *Neighbors* and *Verifiers* to address it.
- The hash of each *EUI* is signed with the private key of the node. It is then transmitted to *W_i_* randomly chosen *Neighbors* as part of the block (Figure 6. Block structure). This way, *W_i_* can verify easily: a. the identity of the transmitting node and b. the validity of the received block.
- *Hashing Mechanism*: SHA-256 algorithm is chosen for binding the blocks. The Arduino Cryptography Library was utilized and tested.
- *Communications Mechanism*: The selected testbed provides an endless range of connectivity options (i.e., Wired Com Ports, Wi-Fi, Bluetooth, Bluetooth Low Energy, analog RF, Fiber Optics, etc.). For our purposes we utilized the build-in wireless capabilities of the device (WiFiNINA library 1.7.0 with 1.4.0 firmware). This allows for fully autonomous peer connectivity *without the need of external devices, access points*, etc., which was a primary red-line. By default, each node is a fully functional Wi-Fi access point (while in “*Listening*” mode) and becomes a Wi-Fi client on the occurrence of the local event.

#### 3.8.2. Testbed

The experiment took place on a setup of two Arduino 33 Nano IoT devices, operating under 1.4.0 framework. NINA library 1.7.0 was used for networking [32]. The specific IoT devices were chosen due to the fact that they satisfy the minimum requirements without the need for external components: They facilitate full WiFi, Bluetooth and BLE connectivity, and support TCP/IP, allowing for peer, fully autonomous operation. They have adequate CPU capacity to perform SHA-256 and RSA, while their internal memory can store hundreds of event blocks.

### 3.9. Security Considerations

The *Witness Protocol* is designed to operate over private P2P connections, without the need for central network access, mainly to increase the non-repudiation immunity of the system.

Ground base scenario: Lets’ consider a set of digital temperature sensors put inside the refrigerators on an industrial facility. Upon thresholds reached, they transmit their readings to readers outside of the fridges. By implementing the *Witness Protocol*, they also peek w peers out of the pool of neighbors as additional witnesses of the event. With respect to each application requirements, the sensitive data of the block can be signed by the owner and encrypted using RSA. Selected events taking place within one device are then transmitted to the selected peers. The system is write-intensive (writes happen at a much-higher rate than disputes).

The *Witness Protocol* runs on top of the casual sensors’ activity and poses no additional privacy concerns. On the contrary, it increases the non-repudiation immunity of the community. In the light of a dispute, the validity of the recordings can be verified at finite cost, either by a peer node, or by an external verifier, by accessing in the witnesses’ local memories.

The *Witness Protocol* falls under the *Byzantine Generals* conceptual frame. The *Byzantine Generals* problem, solved under a wide variety of initial condition assumptions [33], sets the frame in modern consensus vulnerability analysis [34,35,36]. Herein we consider a variation of the BFT in which messages are being constantly broadcasted in a dynamic *Neighborhood* of nodes. The *Witness Protocol* falls under the *BFT with-oral-messages* problem sub-category. The process resembles an “*ear-witness*” real world scenario. To cope with the resources, capacities, and consumption limitations of the IoT devices, WP foresees only one-hop event distribution, no “*gossiping*” and no retransmission of past events.

#### Internal & External Integrity

- Internal Integrity: SHAs’ one-trap door property, suggests that as soon as a block is added in a local chain, the only way to successfully tamper it without being noticed, is to alter the entire part of the local chain from this block to the last ($\frac{M}{2}$ blocks on average). Indeed, a lonely IoT device with hash-linked recordkeeping may be compensated at a trivial $O\left(\frac{M}{2}\right)$ cost.
- External Integrity: As soon as a local event *M* of node *i* (i.e., $N_{i}(M$)) is successfully transmitted to a number of neighbors-witnesses (*W > 1*), there is no way to solely attack $N_{i}$. For the successful tampering of any *Event Block* on any local memory, a combined tampering attack to the “absolute majority” of the set *W* of the witnesses of the event will have to take place concurrently. This leads to a minimum “false quorum” consensus requirement of $\frac{W}{2}+1$ nodes [37,38]. It is noted that this describes the worst-case scenario, in which the node $N_{i}$ under attack has *W* witnesses, out of which no one has witnesses of his own on its’ successive events. It is evident that, time evolving, every node will acquire multiple witnesses at the rate of $\approx \frac{W}{T_{eventperiod}}$, (given random choice of witnesses on each event).
- After a maximum timeframe of $\approx N*\frac{T_{eventperiod}\;}{W}$ all nodes in a neighborhood will become interconnected, introducing $\frac{N}{2}+1$ minimum quorum consensus requirement to the attacker.

## 4. Results

### 4.1. Benchmarking and Findings

A number of processes has been benchmarked, providing the post-processing model with detailed data:

Timestamping:

Time needed for a new event block to be added to the *Blockchain* (micro-nlockchain corresponds to the aggregate of the local chains (depicted in Figure 2 and Figure 7)):

$$T_{teller}=T_{addtolocal}+T_{transmit}$$

Time each node is in *Listening* mode (within an event period):

$$T_{listener}=T_{eventperiod}-T_{teller}$$

Time needed for a node to process and add an event in its *Local Chain*:

$$T_{addtolocal}=T_{sha-256}+T_{sign}+T_{copytochain}$$

Time needed for a listening node to verify add external event in its *Local Chain*:

$$T_{addexternal}=T_{receive}+T_{verify}+T_{addtolocal}$$

Block verification time: $T_{verify}=\left(\#BlockBytes\right)\cdot T_{sha-256}+T_{decrypt}$

Total Block transmission time: $T_{Transmit}=\overset{w}{\underset{1}{\sum }}\left(T_{swapmode}+T_{connect}+T_{serialprint}\right)$

Combined Probabilities are then defined:

Probability of $N_{i}$ being in “*teller*” or “*listener*” mode at a given time:

$P_{i}\left(teller\right)=\frac{T_{teller}}{T_{eventperiod}}$, $P_{i}\left(listener\right)=1-P_{teller}$ respectively.

Any local event may be transmitted at most $W_{effective}$ times before a new local event rises:

$$W_{effective}=\frac{T_{eventperiod}}{T_{swapmode}+T_{listener}}$$

given normal event probability distribution over the *Neighborhood* (i.e., local events are considered to occur over the nodes of a neighborhood in the same rate).

Probability of node $N_{i}$ successfully becoming witness of a specific event is then defined as:

$$P_{i}\left(witness\right)=P_{i}\left(listener\right)-P_{i}\left(iserveothers\right)=P_{i}\left(listener\right)-\frac{\sum_{j=2}^{W_{effective\;}}P_{j}{\left(teller\right)}_{\;}}{N-2}$$

In our variation of the BFT problem-solution, point to point oral messages over *non-gossiping* nodes are considered: nodes do not propagate other nodes’ events, nor they are re-transmitting their own past events.

**Figure 9:**
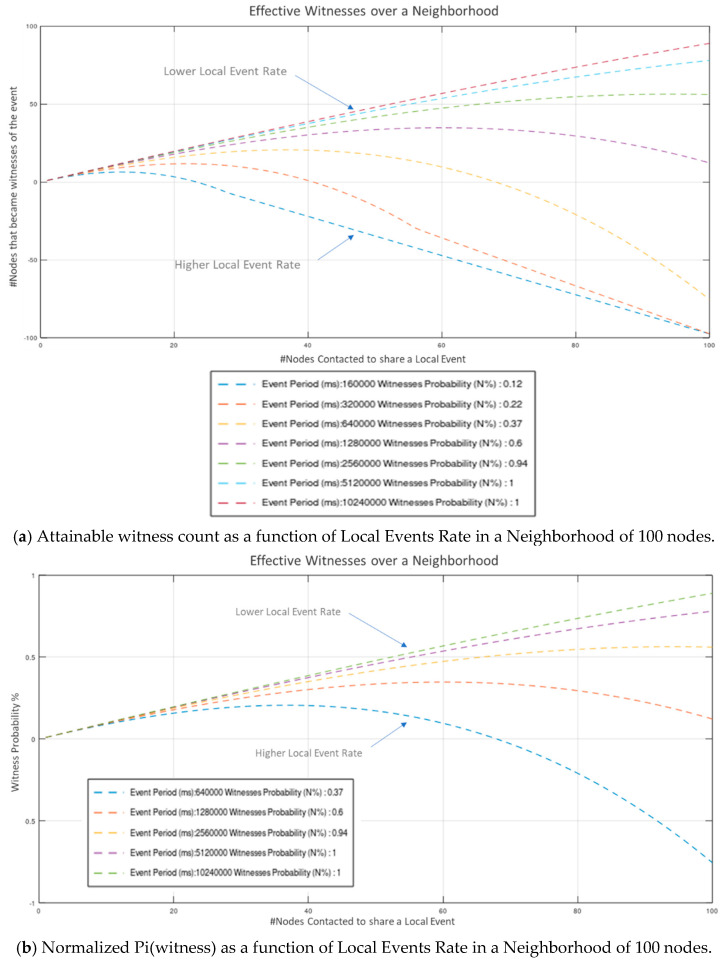
Attainable witness count and Normalized witness probability as a function of Local Events’ Rate in a Neighborhood of 100 Nodes.

**Figure 10:**
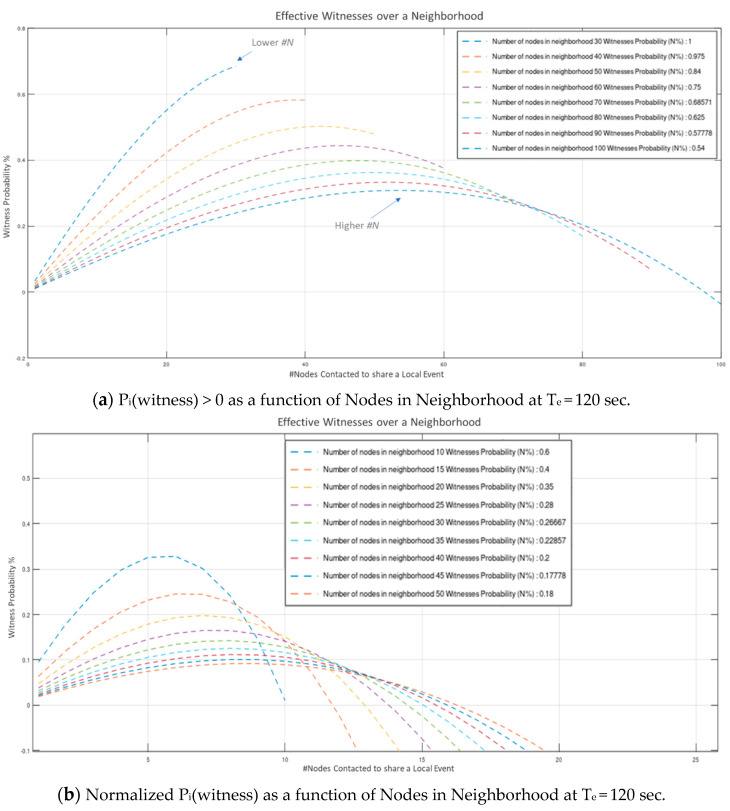
Witness probability and Normalized witness probability as a function of number of Nodes in Neighborhood at Te = 120 s.

Benchmarking recorded the following values (Table 2):

Values indicate that a considerable amount of effort is consumed to constant Wi-Fi connections and disconnections (required to establish fully autonomous peer operation). Even though this overhead is susceptive of significant optimization (via broadcasting, multicasting, multiple concurrent connections, multithreaded processing, etc.), the network proved highly capable of handling neighborhoods of 10-s of nodes in “*human-timescale*” events rates. Results depicted in Figure 9a to Figure 10b indicate that a neighborhood population of up to 64 nodes, operating at an average “*fair*” inter-events time period of 120-sec, can operate without significantly compromising the number of acquired witnesses ($W_{attainable}=N-1)$. Modest communications’ optimization, is estimated to allow for up to 128 node neighborhood (estimation is based on the basic Arduino Nano33 IoT microcontroller performance recorded in the experiment). Further to this, our results verify that for a constant, arbitrarily high neighborhood population, the probability of acquiring *N* effective witnesses rises monotonically with average event period: $\underset{T_{eventperiod}\to \infty }{\lim}\left(P_{i}\left(witness\right)\right)\to 1$, while for a constant $T_{eventperiod}$ the number of witnesses that can be effectively acquired $W(P_{i}\left(witness\right)>0)$ is bounded by the overall event processing time.

We highlight the performance of the system as a function of |W|, i.e., the number of nodes contacted to share local events. We assume equal local events’ rate = $\frac{1}{T_{eventperiod}},\forall N_{i}$ (for all Nodes in Neighborhood).

In Figure 9a, the number of nodes that actually became *Witnesses* as a function of the number of *nodes contacted* on a neighborhood of 100 nodes is depicted for various values of $T_{eventperiod}$.

In Figure 9b, the combined probability of the next-contacted node successfully becoming Witness as a function of *the targeted number of witnesses W* in a neighborhood of 100 nodes is presented.

For a “fair”, human-timescale average $T_{eventperiod}$ of 120 s (period of *new* local events on each peer node), the normalized probability of W nodes effectively witnessing an event ($P_{i}\left(witness\right)>0)$ is depicted on Figure 10a,b s as a function of the neighborhood population respectively.

### 4.2. Data Availability Statement

The core of the source code and the technical data will be made available under Creative Commons license to facilitate future IoT micro-blockchain applications’ development.

## 5. Discussion

Our experiment proves that low-cost low-end IoT devices bear all the necessary capacities in terms of CPU power, connectivity and software tools, to establish and support robust, distributed, blockchain-based operation. The fact is intriguing. It is expected to bring unprecedented security and transparency in the IoT scale, and through this, to the entire transactional activity. The invoked mechanisms are soon expected to become “hardwired” down in the sensors’ bit-level.

Bringing blockchain down to the tiniest operational level, requires in-depth, substantial case-specific considerations and poses significant trade-offs (e.g., the *Witness Protocol*, while optimizing writes, it mandates simultaneous full access to the memory contents of all devices to effectively verify events).

Still, considering the relatively low disputes’ occurrence probability compared to writes’, this is an elegant compromise, which guarantees a significantly high attack cost: O(1) being the cost of altering the contents of an single event block in an M-length random access memory, our architecture demonstrates $O\left(\frac{M}{2}\right)$ attack cost for a “lonely” IoT device and a $O\left(\frac{M}{2}*N\right)$ attack cost to a stationary neighborhood of N nodes.

The perception of *Transaction* itself, was pushed to its’ conceptual limits, to also include unilateral interactions.

Results are intriguing and can be interpreted under a variety of contexts. In a straight analogy to the real-world ear-witness event situation, it is up to the witness to cooperate, unveil identity and provide world with his own version of “*Reality*” (aka his/her memory contents).

The probability of effective witnesses’ existence upon an event, subjects to limitations extensively studied in communication networks’ theory [39]. In our testbed, single-channel communications were considered: at a given time, every node can either *Listen*, or *Transmit*. Swapping from *Listener* to *Teller* required each node to become an active Access Point and back, imposing significant overall delay.

Since disputes are *rare events* compared to the number of the significant events taking place, our micro-blockchain is optimizing “*writes*”. Indeed, writes comes at the cost of O(W+1) both in space and in overall computational volume.

The number of witnesses acquired on each event is subject to a combination of variables, i.e., The number of nodes in the neighborhood, the frequency and distribution of the events, network connection and transmission latency, number of desired witnesses each time, etc.

To compensate the integrity of the *Neighborhood* via the typical 51% type of attack, at least $\frac{W}{2}+1$ nodes have to be compromised by a malicious attacker “*at-the-same-time*”. Our results indicate an optimal maximum W→N. Reads/verifications impose a total cost of $O\left(N*\frac{M}{2}\right)$ and presume concurrent access to the memory contents of every node in the *Neighborhood*.

Irrespective of the communications overhead, a significant amount of CPU and storage capacity must be dedicated in running the chain. This is subject to each application requirements and thorough consideration of the variables and cost-hazard balancing in each case is essential.

## 6. Conclusions

In this paper, the fundamental factors for building, operating and testing micro-Blockchains on the IoT are introduced. The presented experiment reaches down to the individual IoT device in micro-controller level and paves the way for blockchains to get even deeper, becoming functional constructs down to single-sensor and single-processor level.

Blockchain being a software-intensive “conceptual containment” of a series of technologies, from secure hashing, to cryptography, distributed storage, computing and networking, its’ core aspects are highlighted and analyzed. To provide with a solid development framework, the novel *Witness Protocol* was deployed and tested. The setup proved totally capable of processing and securing everyday transactions over neighborhoods of tens of nodes.

The major aspects of the process are presented with direct analogies to the technological frameworks and their philosophical foundations.

Establishing distributed consensus without the need for centralized third-parties is an evolving revolution of our era. Yet, despite of the exceptionally intriguing results from the technical point of view, the current work aspires most of all to pose a small step towards the distributed trust and liability transformation which takes place in societal level on the vehicle of blockchain.

## Figures and Tables

**Table 1:** Indicative minimum IoT device capacity outlining.

Metric	Description	Minimum Indicative Value (Witness Protocol)
Memory capacity	Each Event block is expected to occupy rank 10^2^ Bytes. In *Witness Protocol* an event record of 248 Bytes is utilized. In case of programmable IoT implementation, extra memory space for the program is required. In our implementation, 37.7 KB including all necessary libraries were occupied by the program.	496 Bytes (for 2 records) or M*248 for Local Event chain of length M plus ~40 KB for program storage
CPU capacity	Equivalent capability for executing rank 10^2^ Bytes SHA-256 and 32 bit RSA at a rate range of 10^−5^~10^2^ Hz A write in the Local Chain under the *Witness Protocol* was held on an average of 20 ms time. Communications overhead comes on top and vastly varies	8 MIPS 8-bit AVR family microprocessor is found capable of serving up to ~10^2^ events per second on the local chain (see Section 4 Results)
Data transfer	Single-Event data transfer in WP was benchmarked at 510 ms. Communication speed is setting the overall WP Blockchain operation rate limit.	Event data transfer speed down to 0.5 Kbps were proved adequate for multi-node Neighborhoods of relatively low (human-timescale) event rate (Section 4-Results)

**Table 2:** IoT micro-blockchain benchmarking table.

Parameter	Time (ms) ^1^
$T_{Swapmode}$	3713
$T_{Transmit}$	519
$T_{Receive}$	579
$T_{Verify}$	3
$T_{sha-256}$	4
$T_{copytochain}$	9

^1^ Active benchmark points in source code, utilizing millis() function.

## Data Availability

New data were created and analyzed in this study. Data sharing not applicable.
